# Constructing narratives of heroism and villainy: case study of Myriad's BRACAnalysis^® ^compared to Genentech's Herceptin^®^

**DOI:** 10.1186/gm412

**Published:** 2013-01-31

**Authors:** A Lane Baldwin, Robert Cook-Deegan

**Affiliations:** 1 Genome Ethics, Law & Policy, Institute for Genome Sciences & Policy, Duke University, Box 90141, 304 Research Drive, Durham, NC 27708-0141, USA

## Abstract

**Background:**

The development of Herceptin^® ^is welcomed as a major advance in breast cancer treatment, while Myriad's development of BRACAnalysis^® ^is a widely used diagnostic. However useful and successful this product is, its presence in the public eye is tainted by predominantly negative press about gene patenting and business practices.

**Discussion:**

While retrospection invites a sharp contrast between Genentech's triumphal narrative of scientific achievement and Myriad's public image as a controversial monopolist, a comparative history of these companies' products reveals two striking consistencies: patents and public discontent. Despite these similarities, time has reduced the narrative to that of hero versus villain: Genentech is lauded - at least for the final outcome of the Herceptin^® ^story - as a corporate good citizen, Myriad as a ruthless mercenary. Since patents undergird both products yet the narratives are so different, the stories raise the question: why have patents taken the fall as the scapegoat in current biotechnology policy debate?

**Summary:**

A widely publicized lawsuit and accompanying bad press have cast Myriad as a villain in the evolving narrative of biotechnology. While the lawsuit suggests that this villainy is attributable to Myriad's intellectual property, we suggest through a comparative case study that, at least in the Myriad case, it is not simply about the patents but also other business strategies the company chose to pursue. Patents were a necessary but not sufficient cause of controversy.

## Background

### Introduction

On 29 March 2010, Judge Robert Sweet of the United States District Court for the Southern District of New York shocked the world of intellectual property law with his ruling in *Association for Molecular Pathology v. US Patent and Trademark Office *(the 'Myriad' case). He ruled that Myriad Genetics' patents on the *BRCA1 *and *BRCA2 *genes claimed non-patentable DNA molecules and methods [1]. Attorneys Dan Vorhaus and John Conley wryly observed, 'pigs fly,' [2] at least for awhile in the District Court. Meeting the same fate as the mythical Icarus, the wings constructed by Judge Sweet melted under the scrutiny of the Court of Appeals for the Federal Circuit (CAFC) on 29 July 2011 [3], and were argued again before CAFC on 20 July 2012 by order of the United States Supreme Court. The case was appealed again to the US Supreme Court on 25 September 2012, and certiorari was granted on 30 November 2012 [4-6]]. The case will be heard by the Supreme Court on April 15, 2013 with a decision before July.

Through the eyes of patent practitioners, Judge Sweet's decision was an anomaly, but it is just another episode in shifting jurisprudence, with a succession of cases between CAFC and the Supreme Court. This case could become another decision that narrows the scope of patent protection. Indeed, it already has, with Myriad's broad method claims being invalidated by both the district court and CAFC. Backed by a decade of precedent patenting genes, the patents that Myriad Genetics holds on *BRCA1 *and *BRCA2 *genes continue a long-standing pattern of granting similar patents in the United States [7]. Accounts of the gene discoveries widely acknowledge that aside from contribution to science and society, patents and publication were the brass rings to be grabbed by contenders in the great race of 1990 to 1995 to identify, clone, and sequence *BRCA1 *and *BRCA2 *[8-11]]. While there have been gene patent controversies over the years, none has approached the intensity of public conflict over *BRCA *patents [5]. Even before the current litigation began, policy reports around the world cited *BRCA *far more often than any other gene patents [12], and public news media coverage is far more extensive for *BRCA *than other gene patents cases (most of it strongly negative coverage) [13].

Why have these particular patents aroused such intense controversy? As patent scholar Rebecca Eisenberg noted, 'Significant opposition to gene patenting within the medical and scientific communities did not arise until the patentability of DNA had long been established' [7,14]. It may be helpful to assess whether the controversy should properly be attributed to patents themselves, or to unpopular business practices that Myriad could put in place because of patent exclusivity that made it the only US commercial *BRCA *testing service. Should the focus be on whether Myriad should have gotten patents at all, or also on what Myriad did with them?

To assess the extent that the patents played a role in the malcontent amassed against Myriad, we selected a comparable story of product evolution as a point of comparison: Genentech's Herceptin^®^. While not directly comparable because Herceptin^® ^is a therapeutic and BRACAnalysis^® ^is a diagnostic, the development of Herceptin^® ^nonetheless resembles that of BRACAnalysis^® ^in several respects. Both are novel breakthroughs in managing breast cancer. Both were brought to market by biotechnology companies. Both products were mainly developed in the 1990s. Most importantly, for the purposes of this analysis, both inventions were patented. Among the differing elements of these two stories is profoundly different public reception. Instead of a pubic outcry in the form of a very public lawsuit, Genentech was celebrated with a corporate leadership award from the National Breast Cancer Coalition [14]. Thus, to answer the question of what role patents have played in public perceptions of Myriad, we have sought to compare Myriad's corporate history with Genentech's development of Herceptin^® ^with significant patent protection on a novel product, which encountered a very different reception from a similar constituency at more or less the same time.

### Methods

To assess the question posed in our thesis, we compared Myriad's development of BRACAnalysis^® ^to Genentech's Herceptin^®^, in order to assess the role of patents relative to other factors, such as engagement of the constituencies most directly affected (people at risk of developing breast and ovarian cancer) and compliance with health professional standards and norms. We constructed this history by surveying the relevant literature, US Securities and Exchange Commission (SEC) reports, corporate statements, media reports, patent databases, and off-the-record interviews with relevant actors. In this analysis, we attempt to isolate patent and non-patent factors, describing how Myriad and Genentech developed their respective products. This includes differences between therapeutics and diagnostics, how professional guidelines were treated, and most importantly how the companies dealt with intense controversy among breast cancer constituencies. One crucial difference is how the companies dealt with the nationally recognized and well-organized advocacy organizations when conflict with those organizations erupted. We will first provide a basic historical overview of how BRACAnalysis^® ^and Herceptin^® ^were developed, looking for factors that might explain the divergent narratives.

## Discussion

### Corporate histories

#### Myriad Genetics

On 17 October 1990, geneticist Mary-Claire King, then at the University of California, Berkeley, made a groundbreaking announcement to the American Society of Human Genetics: her team had discovered a genetic linkage to breast and ovarian cancer on chromosome 17 [10]. This followed a strategy of locating a gene on the chromosomes by studying families with an inheritance pattern suggesting mutations in a single gene. She found the linkage by comparing multiple affected and unaffected members in such families. The strategy was pioneered by finding a genetic locus associated with risk of Huntington's disease on the tip of chromosome 4 in 1983 [15]. Thereafter, several 'genes for' cystic fibrosis, Alzheimer's disease, neurofibromatosis, and other conditions were identified by cloning and sequencing DNA from the region and identifying disease-associated mutations [15-17]]. King extended the strategy to breast cancer, not then commonly considered a 'genetic' condition. She focused on families that had many members affected by breast cancer at a young age, suggesting a broken gene might be found. It turned out that ovarian and some other cancers also traveled in these families, in a pattern consistent with inheritance of a single mutated gene increasing risk of both ovarian and breast cancers. King and her colleagues thus located but did not clone and sequence the gene. "King's discovery was like tracing the correct address to the confines of New York City," the starting flare for an intense race to clone and sequence the gene by finding disease-causing mutations [10]. It was often characterized as a hunt for the 'breast cancer gene', but it was not quite that. Rather, it was a hunt for mutations that altered a gene whose biological function was entirely unknown, but the consequence of its mutation was predisposition to certain cancers. The race lasted four years, fluctuating between intense international competition and periods of team collaboration.

As the search dragged on, the incentives of pride, patents, and publication were leavened by diagnostic hope - being able to unmask the silent hereditary predator that had devastated and stigmatized generations of women. The contenders were among the biggest names worldwide in human genetics research and, in the end, it was the team working with Mark Skolnick of Myriad Genetics that uncovered the *BRCA1 *sequence by finding cancer-associated mutations. In August 1994, Skolnick announced that he had uncovered the *BRCA1 *gene [10]. Earlier that month, another contributor to hereditary cancer had been unmasked when a putative *BRCA2 *gene was tentatively located on chromosome 13, by Michael Stratton and his team of UK scientists [18]. Stratton's results identifying this sequence as the *BRCA2 *sequence were published in *Nature *in 1995 [19]. In 1996, Myriad's team, arguing that Stratton's published sequence was incomplete, followed this publication with the complete sequence and a more extensive mutational analysis [20]. That resulted from another race that culminated in cloning and sequencing mutations in *BRCA2 *in 1995 [10]. The *BRCA2 *race ended in a dead heat between a group led by Michael Stratton in the UK and the Myriad/Utah team in Utah [21,22].

Myriad's work was partially funded by government grants to the University of Utah. Some work was done by government scientists at the National Institute for Environmental Health Sciences, and some was done by Myriad itself, using funds from its investors and under an agreement with Eli Lilly & Co. Myriad thus co-funded the work, and it was no secret that patents would be sought. And patenting was not confined to Myriad, Mary-Claire King's linkage method was patented and licensed to OncorMed by the University of California, and OncorMed itself had a US patent on *BRCA1 *(see below).

Myriad filed its first *BRCA1 *patent applications in August 1994, and this initial application ripened into several patents, starting with the grant of its first *BRCA1 *patent, 5,693,472 in 1997 [10,23] OncorMed already had a *BRCA1 *patent covering the consensus wild-type sequence [24]. In 1998, OncorMed sued Myriad and then Myriad counter-sued for patent infringement. This case ultimately settled, with rights going to Myriad under a confidential agreement [9]. The patents originally assigned to OncorMed and Gene Logic have since been re-assigned to Myriad.

Using these patent rights, Myriad Genetics became the sole commercial testing service for mutations in *BRCA1 *and *BRCA2 *in the United States. A patient carrying a mutation in these genes can have up to an 87% risk for developing breast cancer and a 44% chance of developing ovarian cancer in a lifetime [25]. Studies have shown that patients with deleterious mutations can reduce their risk of cancer through prophylactic measures such as taking tamoxifen or other chemopreventive agents, or surgical removal of breasts and ovaries [26,27].

Armed with intellectual property protection on both compositions of matter (claims on isolated DNA molecules) and diagnostic methods, Myriad developed its BRACAnalysis^® ^test [28]. It is the first-line diagnostic in high-risk families with early-onset breast and ovarian cancer, with second-line testing for major rearrangements (Myriad's BART^® ^test) or sequencing 20 other genes for mutations in other genes that are much less common but can also confer inherited cancer risk (tests available from Ambry Genetics and academic laboratories). BRACAnalysis^® ^is Myriad's flagship product, accounting for over $400 million in revenue and 80% of Myriad's revenues in 2011, with BART^® ^and other tests for inherited risk of pancreatic cancer, colorectal cancer, melanoma and other tests accounting for most of the rest of Myriad's revenues [29].

Clinicians and patients alike readily acknowledge that Myriad performs very well in doing tests, reporting results, marketing, and obtaining reimbursement from third-party payers. Myriad is quite proficient and competitive with other genetic diagnostic companies [24]. However, in the years since Skolnick's impressive discovery, the corporate image of Myriad Genetics has consistently been unpopular. Caulfield, Bubela, and Murdoch reviewed English-language newspaper articles on Myriad Genetics and the *BRCA *gene patents in many countries [13]. Not only did Myriad's gene patents garner more international news attention than other gene patenting controversies, but also 77.6% of these articles had a negative tone.

This presents an anomaly: a company that is widely regarded as being an efficient laboratory, was a startup that helped discover the genetic cause of two dread cancers and provides a service that allows people at risk to mitigate the risk is nonetheless reviled. This should be a hero story but is instead a dark narrative. Why? The contrast with Genentech and Herceptin^®^, a product that developed at more or less the same time and for an extensively overlapping customer base, is striking.

#### Genentech

The beginning of the Herceptin^® ^story begins not at Genentech, but at the Massachusetts Institute of Technology's Whitehead Institute, where Robert Weinberg discovered the gene that led to one of the most significant discoveries in breast cancer therapeutics [30]. However, *neu *(christened for its original discovery in rat brain tumors) was found when looking for cancer-associated genes, when studying basic molecular and cellular events associated with cancer; its clinical significance beyond advancing basic cancer biology was not immediately apparent. It was not until 1989 that Axel Ullrich, Dennis Slamon and colleagues published the relevance of the *Her-2*/*neu *gene (re-christened in deference to Slamon, Ullrich, and Weinberg's work) [31]. Over-expressing *Her-2*/*neu *was associated with a particularly nasty form of breast cancer. One strategy to address this was to find an antibody that would bind to the *Her-2*/*neu *protein in hopes of inhibiting its effects, thus countering its overexpression. The promise of potentially curing women with an aggressively recurring type of breast cancer without the 'kill, burn, and slash' combination of chemotherapy, radiation, and surgery was eventually realized in Herceptin^®^, the resulting therapeutic. The monoclonal antibody inhibiting *Her-2*/*neu *was patented and manufactured by Genentech, which has continued to produce it as Herceptin^®^.

Genentech's management of Herceptin^® ^did not always garner good will among national breast cancer advocates. Indeed, Genentech got off to a rocky start, as it was reluctant to plow resources into a monoclonal antibody product, given the many disappointments from monoclonal antibody therapeutics over the previous decade [30]. And at the time, cancer was not widely regarded as a promising target for 'blockbuster' drugs, especially when this proposed product would be for the minority of those with breast cancer who showed *Her-2*/*neu *over-expression. Slamon was a constant clinical champion, and apparently also sometimes regarded as a thorn in the side of Genentech senior management [30].

Women from Breast Cancer Action (BCA) in San Francisco demonstrated actively outside Genentech headquarters to pressure the company into providing access to the then-experimental treatment. Genentech contacted Fran Visco from the National Breast Cancer Coalition (NBCC), a national umbrella advocacy organization. She agreed to help develop the clinical studies needed to show clinical safety and efficacy, so long as NBCC and other authentic patient representatives were fully engaged and 'at the table' in helping design and carry out the trials [30]. In no small part because of NBCC's standing as a highly credible national advocacy organization, the trials needed to generate data for Food and Drug Administration (FDA) approval proceeded apace.

The initial clinical trials were quite promising, and despite several fits and starts by Genentech and its then-partial owner Roche, Herceptin^® ^received FDA approval in 1998. As a specific therapy, Herceptin^® ^was only effective on the 25% of tumors that expressed high levels of *Her-2*/*neu *[32]. While it was not effective for most breast cancers, many patients who responded to it improved dramatically. In early clinical trials, patients with advanced disease lived 5 months longer when treated with Herceptin^® ^and chemotherapy [32]. A 2005 *New England Journal of Medicine *article reported that Herceptin^® ^reduced relapses by half in women who started treatment early [33]. Herceptin's success was so widely celebrated during its early clinical trial stage that Genentech was deluged with demands for a compassionate access program to provide the drug for terminal patients looking for a last chance at therapy [30].

In 2009, Roche's partial ownership of Genentech became full acquisition, combining Genentech's operations into Roche's [34]. In the last full year before this acquisition, a January 2009 SEC filing reported that Genentech generated $9.5 billion profit from US product sales out of operating revenue of $13.4 billion. Herceptin^® ^sales accounted for $336 million of those revenues in 2008 [34,35]. In 2011, global Herceptin^® ^sales reached $5.728 billion [35].

*BRCA *testing and Herceptin^® ^had parallel trajectories: both contributed novel insights into breast cancer and had clinical implications that demonstrably improved survival; both were brought to market by start-up biotechnology firms; both staked patent rights as part of the business model; and both products became significant profit centers. These common factors belie dramatic differences in the public reception accorded these two products. The contributing differences lie in each company's corporate practice and in the nature of the products they were developing. In the case of Myriad's reputation, ill will is arguably due less to the patents Myriad acquired than the way it deployed them.

### Assertion of patent protection

The strength and extent of intellectual property protection is largely determined by the enforcement of granted rights. Herceptin^® ^and BRACAnalysis^® ^demonstrate the broad spectrum of patent use, illustrating how the protection of intellectual property plays out in public debate. Once it secured patents, Myriad was quick to pursue aggressive enforcement actions against public and academic institutions both in the United States and internationally. Two particularly salient examples were conflicts with the University of Pennsylvania and the Canadian Province of Ontario.

In her 2009 declaration before the United States District Court for the Southern District of New York, University of Pennsylvania researcher Dr. Arupa Ganguly recalled how in May of 1998, she and her colleague Dr. Haig Kazazian received a notification letter from the Myriad Genetics Director of Corporate Communications acknowledging Myriad's five existing *BRCA1 *patents, some of which were infringed by Ganguly and Kazazian's ongoing *BRCA1 *research (which included *BRCA *testing) [36]. Per Ganguly's recollection, this letter offered a collaboration license that was 'of very limited scope, as it would not allow us to complete diagnostic testing services for *BRCA1*, or comprehensive research on the *BRCA1 *gene which we had been doing at the lab' [36]. After this original notification letter, a flurry of escalating communication continued among Myriad Genetics, Ganguly, Kazazian, and the legal counsel for both parties. Despite researcher attempts to comply with Myriad's original request, increasingly aggressive letters arrived at Penn, demanding written assurance that *BRCA *testing had stopped. As Ganguly recounted, 'I was compelled to cease all *BRCA1 *and *BRCA2 *testing, whether for clinical or research purposes' [36].

In this declaration, Ganguly also recalled another instance of aggressive enforcement letters when working on a research project sponsored by the National Cancer Institute. As an investigator on this project, she provided *BRCA1 *and *BRCA2 *screening to participants. In September 1998, the National Cancer Institute (NCI) received a letter from Myriad Genetics concerning patent infringement by clinical research that involved *BRCA1 *and *BRCA2 *testing. Penn was to serve as a testing core for several NCI-funded trials that included *BRCA *testing and reporting. Ganguly stopped performing *BRCA *testing for the project after the Myriad letter. NCI signed a 1999 Memorandum of Understanding that gave Myriad rights to do *BRCA *testing for NCI-sponsored research unless it did not entail returning results to those tested, or it was done only by laboratories at the same institution as was providing patients' clinical care [37]. In return, Myriad deeply discounted the price to NCI and its grantees.

The Pennsylvania researchers assumed sanctuary from patent infringement under a research exemption [9]. The US research exemption, however, does not include 'research using' an invention, as opposed to a very narrow judge-made case law exemption for 'philosophical' inquiry or 'research on' how the invention works [38,39]. Myriad clearly disagreed that Penn's work warranted an informal research exemption, and Penn's use almost certainly did not qualify for the formal legal exemption. Myriad sent threatening letters to the offending parties. Though the company confirmed it had no intention of enforcing the patents against researchers, the scope of their voluntary forbearance from enforcement was apparently restricted to basic research, not clinical research that entailed testing and reporting results as part of a clinical study, even a federally funded one.

Myriad did not make a public policy statement, but instead conducted the process through legal negotiation and private correspondence. This is common in business-to-business patent conflicts, but it was startling to clinical researchers who viewed their work using *BRCA *testing as a natural outgrowth of their clinical research. In short, this style of operating through lawyers with Penn and other academic institutions that received Myriad's letters was regarded as legalistic bullying. Instead of countering its poor public image, Myriad let the fear persist [9]. Gold and Carbone's case study of Myriad noted, 'to the large majority of researchers who had not been following closely Myriad's public statements, it seemed that Myriad was willing to block scientific research to turn a profit' [9]. It was a short step from this legal maneuvering to a narrative of dark villainy.

When Myriad worked to expand its business abroad, it encountered the Canadian Province of Ontario. In an attempt to take the BRACAnalysis^® ^test abroad, Myriad licensed Canadian test rights to MDS, a Canadian firm. The intention was for Myriad to focus on its practice in the United States while MDS acted as an ambassador to the Canadian provincial governments.

MDS and Myriad encountered strong resistance in the Ontario Ministry of Health. In the Canadian public healthcare system, Myriad's commercialization model and the sheer expense of the test were deemed incompatible with Ontario's genetic services model. Provincial health programs already administered genetic tests and had the information needed to do *BRCA *testing, based on the published sequences and other medical literature. (Such publications included Myriad and Utah papers, but also data from many other groups.) While the governmental policy arm worked to devise a system that could prevent the incorporation problems experienced in Myriad's other international ventures (including the UK and Australia), Myriad grew increasingly suspicious and sent out cease-and-desist letters in May 2001.

The lack of communication between Myriad and Ontario was a misstep on both sides. The rising storm appeared to have quelled in fall 2001, when Ontario's Minister of Health and the President of Myriad Genetics agreed to meet. Myriad's next step seriously escalated the conflict. Myriad presented a package of letters from current and former US government representatives and the Biotechnology Industry Organization, threatening actions as aggressive as 'trade sanctions' and cancellation of the Biotechnology Industry Organization's annual convention in Toronto [9] (the authors of this paper cite these letters, which they have on file). This contretemps resulted in a storm of negative media accounts, marring Myriad's already unattractive public face. It also put any Canadian politician in an impossible bind - push back against Myriad or bow before an American corporation whose demands perturbed practices in the highly popular Canadian provincial health systems.

The media outcry overly simplified the conflict and reduced it to patenting, thus conflating Myriad's poor political strategic decisions and its particular service-monopoly business model with the granting of *BRCA *gene patents. Though Myriad's patent protection did give it the exclusive rights it used this dispute, the threatening behaviors exhibited by Myriad were not inherent to the patents. Indeed, it is hard not to regard Myriad's Canadian gambit as counterproductive, given that Myriad failed to secure the Canadian market (Quebec and some other provinces do refer some tests to Myriad, but most testing is not through Myriad) and also never followed up on the threats. It lost business and destroyed its public image. The patent strong-arming failed. Myriad's decision not to sue or otherwise enforce its Canadian patents further may have been in part because even if it won the patent battle, it would still have had to get coverage and reimbursement decisions from the very provincial health plans it would have to sue for patent infringement. By fighting the Ontario Health Ministry, it was engaging its largest potential Canadian customer. Even an uncertain victory over patents would still face a potential battle over coverage and payment.

An aggressive patent enforcement strategy is not novel as a business use of patents. Patents, after all, are exclusive rights fully intended to forestall competition, and enforcement letters are common among competitors. The distinctive element here was not the act but practices standard in business being applied to researchers and laboratories at nonprofit, government, and academic institutions, and then against a provincial health service. In one sense, this was a standard way to 'clear the market' of competitors, but the nonstandard feature was that the competitors were not competitor firms, and some of the uses were tightly linked to clinical research. This behavior lies at the heart of ill will towards Myriad, especially in the scientific community. As Jim Watson stated in 2010 at the Genomes Environments and Traits Conference, 'I hate Myriad the way some people hate Goldman Sachs' [40]. However standard it might have been to enforce patents aggressively, the resentment generated by this initial patent enforcement planted the seed for a public interest lawsuit a decade later.

### Standards of corporate practice

After Myriad published its discovery of the *BRCA1 *gene, the logical next step was to develop a diagnostic test. Naturally, just as there was a race to find the gene, there was competition to develop the test for that gene. The primary company contenders were OncorMed and Myriad Genetics, due to dually issued patents on properties of the *BRCA1 *gene. The licenses ultimately ended up in the possession of Myriad, but for a brief period both firms had intellectual property and offered commercial *BRCA *testing, giving us the opportunity to retrospectively analyze the divergent corporate practice models, clarifying the elements of Myriad's business model that differed from OncorMed's and that tarnished Myriad's public image.

In March 1996, the Federal Task Force on Genetic Testing published a report making recommendations on the regulation of genetic testing [41]. Among the most controversial elements of this report was the position that regulation of testing centers did not suffice to assess the clinical validity of utility of genetic tests [41]. The report recommended expanding the regulatory criteria under the Clinical Laboratory Improvement Amendments of 1988 (CLIA), the statute that governs laboratories. Approval by a regulatory agency was recommended to ensure quality of informed consent, genetic counseling, and test utility, not just whether the test measured what it claimed, but how and whether it affected clinical decisions and improved outcomes. The two companies, Myriad and OncorMed, took opposite approaches to respecting these recommendations in their *BRCA *test-marketing strategies.

OncorMed was the first to market the *BRCA *test commercially in 1996 [41]. Its commercialization strategy complied with the Task Force's recommendations, including testing only in research protocols approved by institutional review boards. To ensure minimal consumer risk, testing was accompanied by consumer education and informed consent. Pre- and post-test genetic counseling was required, and marketing was directed to physicians participating in research. OncorMed also had very strict family risk criteria that women in pursuit of testing would need to meet in order to avoid undue psychological, emotional, or financial risk, and avoid over-utilization of an expensive test that low-risk women did not necessarily need [41]. OncorMed was publicly committed to introducing *BRCA *testing through a pathway that complied with health professional standards and only in the context of clinical research to demonstrate safety and clinical utility.

Myriad took a different approach. Myriad marketed its test outside research protocols, eventually including direct-to-consumer advertising. Myriad had guidelines rather than strict requirements about who was eligible for *BRCA *testing, unlike OncorMed's strict protocols. Finally, Myriad, 'did not refuse to test based on inappropriate patient selection, did not require a copy of signed consent, and did not require verification of the availability of qualified counselors to assist the patient or that counseling had taken place' [41]. Thus, unlike OncorMed, Myriad did not pledge to introduce *BRCA *testing only through a research pathway that would produce evidence of clinical utility through collaborations with public research institutions.

Clearly the two companies approached the introduction of *BRCA *testing from different perspectives. While OncorMed drove their marketing strategy to meet the requirements of health professional recommendations, Myriad clearly had the desire to reach a broader population. There was a clear benefit to Myriad's approach: the more women who are tested, more data could be collected to better refine and interpret the test, and more women could potentially learn about predisposition to a life-threatening disease. While there is nothing inherently reprehensible about this approach, it was widely interpreted as Myriad's emphasis on profit and lesser commitment to creating an evidence base before widely introducing *BRCA *testing.

Returning to the Herceptin^® ^story, Genentech abided by standards of professional practice by following a strict FDA-approved protocol when introducing its new treatment. However, Genentech had little choice. This is because Genentech was developing a therapeutic product, not a diagnostic service. As the developer of a therapeutic, Genentech had no choice but to conduct premarket-approval studies under strict FDA-approved protocols. Myriad and OncorMed had a choice, because laboratory-developed tests were not subject to FDA premarket approval. Whether right or wrong, ignoring the public statements of advocacy organizations, recommendations of a federal task force, and *BRCA *testing guidelines of several health professional groups did little to ameliorate Myriad's image as a profit-maximizing renegade.

In developing a diagnostic for risk assessment rather than a treatment for cancer, Myriad was arguably already at a disadvantage in the quest for public opinion. As crucial as BRACAnalysis^® ^has been to the advancement of breast cancer management, it simply provides information as opposed to treating a disease. Where Herceptin^® ^has the power to rid a woman of her cancer, BRACAnalysis^® ^has the power to affirm the probable occurrence of a woman's cancer, or identify the genetic cause of an existing cancer. This favors a more welcoming reception for Herceptin^®^, because a therapeutic is more directly connected to saving a life.

In the US healthcare system, the issue of access to expensive drugs was widely understood to be a problem, but a common one for therapeutics and medical devices. However, BRACAnalysis^® ^was similar to a routine blood test diagnosing the presence or absence of a condition. In fact, Mark Skolnick commented that, "there's no difference in my mind or in the government's mind between a lipid assay, a protein assay, an immunoassay, or a DNA assay" [41]. The profit margins were generally lower for diagnostics, and access problems and cost were unusual, although there were also debates about expensive imaging technologies. But applying the 'blockbuster' financial model - charging high prices for a patented product - was novel for a diagnostic. While this test is under the same functional taxonomy as a routine clinical test, patients found themselves confronting barriers such as cost and access more often associated with a pharmaceutical drug or expensive device.

The economics of product development are also quite different between diagnostics and therapeutics. Genentech had to conduct complex and very expensive clinical trials to prove clinical safety and efficacy before marketing Herceptin^®^, but the expense of developing a *BRCA *test was considerably lower. Myriad also invested in a testing laboratory, certification of its procedures, and incurred expenses in developing BRACAnalysis^®^. Much of this investment was, however, attributable to its particular sole-source service model, not necessarily to developing *BRCA *testing. It could, for example, have out-licensed the test. There were already nine laboratories offering *BRCA *testing that withdrew from the market when Myriad enforced its patents [42]. Some of these were university or nonprofit testing laboratories (such as Penn or Mayo). That is, while no university could develop, test and manufacture Herceptin^® ^except through an industry partner, many laboratories could and actually did introduce *BRCA *testing before Myriad cleared them from the market to establish dominance for BRACAnalysis^®^. They cleared the same barriers to entry that Myriad faced. Thus, although BRACAnalysis^® ^and Herceptin^® ^might have been comparable in some ways, they occupy two very different niches in healthcare services, contributing to different public acceptance.

### Comparative relationships with patient and practitioner advocacy groups

Another contrast between Genentech (and OncorMed) and Myriad Genetics was outreach to and collaboration with advocacy groups representing their potential customers. While collaboration and communication between providers and patient populations is essential to clinical research and medical care, successful corporate behavior is not always determined by the strength of a relationship with representative groups of target consumers. When a business monopoly is created, the necessity for positive corporate relationships with consumers is diminished, as there is no competition. A comparative analysis of advocacy group interaction between Genentech and the National Breast Cancer Coalition during Herceptin^® ^development, and between Myriad Genetics and many patient and practitioner advocacy organizations in the introduction and marketing of BRACAnalysis^® ^reveals a crucial discrepancy in how the companies managed constituency relationships, especially after initial opposition was encountered.

#### Myriad Genetics

Given Myriad's focus on hereditary breast cancer diagnostics, a natural ally would be the prominent advocacy group FORCE (Facing our Risk of Cancer Empowered). FORCE was specifically organized to address the needs of people facing inherited risk of cancers of various types, and breast/ovarian cancer accounts for most of its membership. Though FORCE is listed as a reference organization on Myriad's BRACAnalysis^® ^information site, the relationship between Myriad and FORCE has become strained over time [25].

Two representative instances of Myriad's handling of advocacy organizations are: (1) in public commentaries condemning Myriad's use of its patents; and (2) concern about Myriad's marketing of tests direct to consumers and to primary physicians without the professional standard of genetic counseling. In a statement to the United States Patent and Trademark Office, FORCE Director Sue Friedman wrote that, 'We believe that the exclusive gene patents of the *BRCA1 *and *BRCA2 *genes held by Myriad Laboratories have had a detrimental impact on the community we serve' [43]. This statement continued to elaborate on three primary issues with the patents: the stifling of research, a negative impact on test interpretation, and high cost. FORCE is correct in asserting that these corporate issues are related to Myriad's gene patents, but an important point to make is that patents do not inherently produce these kinds of behaviors. If *BRCA *testing had evolved from discoveries and patents held by Mary-Claire King, Michael Stratton or some of the other competitors in the race to find *BRCA *genes, it is possible that licensing and business plans would have generated far less intense opposition, possibly even if licensed exclusively to a firm that allied itself with breast cancer organizations and followed the course that OncorMed initially pursued [41]. Collaborating with advocacy groups like FORCE or NBCC or BCA when making decisions about testing policy might have changed the underlying story by changing the perceptions and behavior of key constituency organizations.

Another example of controversial behavior is Myriad's decision to stop contributing to the Breast Cancer Information Core (BIC) database in late 2004. The BIC database is a public database of breast cancer susceptibility variants encountered in clinical practice and research. This information is made available to qualified investigators to improve clinical understanding and enable more effective clinical interpretation of *BRCA *variants [44]. Myriad contributed to BIC into 2004, but then stopped doing so, initially because of technical issues, but by 2006, it was a deliberate strategy to build a database that would leverage the company's large testing experience into a proprietary database that would not expire with Myriad's patents. By withholding these variants from the public database, Myriad gained a competitive trade secret advantage over other companies. The trade secret database will persist when their patents run out, and will remain an advantage until public data sources supply the same information [45]. FORCE notes in a 2010 *Genomics Law Report *article: "Among other things, such a strategy would run contrary, at least in sprit, to a policy against extending patent monopolies beyond their terms" [28].

The proprietary database is trade secrecy leveraged on patent monopoly, and it is perfectly legal. By becoming the world's largest testing service, Myriad also discovers new variants and incorporates those into its database. The data are generated at Myriad's expense (albeit from payments for clinical services on samples sent for genetic testing). For other genetic conditions, clinical interpretation is largely based on public data; for *BRCA *testing, Myriad has a distinct advantage, even over the best academic centers, because of its unique data set. Again, this is neither illegal nor illogical; but it is a novel practice in clinical genetics, and so new for this constituency. Leveraging a proprietary database from a patent-based monopoly seems likely to generate controversy as awareness of this practice grows.

On a similar note, FORCE has criticized Myriad's failure to develop a promising therapeutic. In her 2012 statement to the US Patent and Trademark Office (USPTO), Friedman addressed problems in dealing with Myriad when developing drugs to treat breast cancer. Poly-ADP ribose polymerase (PARP) inhibitors are a promising class of drugs for cancer treatment. Cancers with mutations in *BRCA1*/*2 *may be particularly promising to study. In order to develop this class of drugs, the companion laboratory that develops the drug needs to also gain FDA approval for a diagnostic [43]. Though the most recent policy statement from FORCE [46] does not bring up this point about therapeutic development and Myriad has announced it is indeed working on companion diagnostics for at least one PARP inhibitor's manufacturer, from its prior testimony it is clear that Myriad's CLIA-certified *BRCA *test was crucial for development of the therapeutic, and yet licensing for drug development was problematic for at least some manufacturers, and a concern to FORCE [47]. [48]Again, this is a perfectly legal and understandable business behavior, and a completely foreseeable consequence of exclusive rights, but it does generate controversy and could impede advance of a therapeutic approach [47].

Test cost and efficacy are also a source of concern to advocacy groups. In its statement to the USPTO, FORCE cited limited financial assistance and an increase in test cost over time, despite dramatic drops in the cost of DNA sequencing. The Myriad test 'list' price out-of-pocket is in the range of $3,300, somewhat higher than its price a decade ago, with an additional $700 for expanded BART^® ^testing if needed (pricing quoted by a Myriad customer service representative to one of the authors [ALB] on 7 August 2012), which some define as the 'standard of care' [49]. In her *amicus *brief to the United States District Court for the Southern District of New York, Dr Elizabeth Swisher, an oncologist, noted, "approximately one-third to one-half of my patients for whom I request genetic testing do not meet Myriad's criteria; yet, I think most should receive this additional [BART^®^] genetic testing" [49]. She also pointed to an article in *Cancer Research *recommending BART^® ^rearrangement testing as standard of care for high-risk women, including those outside of Myriad's screening criteria [50].

The Yale Society of Genetic Counselors, an advocacy group of physicians, nurses, and genetic counselors associated with Yale, has been particularly outraged by the additional cost associated with BART^®^. This group posted a public open letter imploring the company to incorporate the BART re-arrangement screening into its comprehensive BRACAnalysis^® ^test, instead of treating it as an extra-cost test conducted only when BRACAnalysis^® ^is negative and yet suspicion of a *BRCA *mutation persists [51]. CNN covered the letter in an October 2011 news story [52]. Yale genetic counselor Ellen Matloff stated, "What Myriad's doing - charging extra for this test - is really sleazy. They're collecting blood money off my patients." This inflammatory quote is representative of the extent to which some have been alienated by Myriad's business practices. Many businesses encounter strident language questioning their practices (for example, many groups have questioned Genentech's pricing of Avastin^®^, and NBCC applauded FDA's July 2010 withdrawal of approval for its use in breast cancer, an indication Genentech fought hard to keep) [53]. The intensity and regularity of public conflict with Myriad, however, appear to be part of its DNA.

This poor communication with relevant advocacy groups over BRACAnalysis^® ^started in the wake of the gene's discovery, before the test had even been fully developed. In 1996, the American Society of Clinical Oncology released a set of guiding principles regarding the management of *BRCA *testing [54]. In response to these recommendations, Frances Visco of NBCC lauded the exciting scientific discovery, but expressed concern that public policy and medical knowledge had not kept pace with the science, and testing should be based on evidence of clinical utility [55]. *BRCA *testing should, therefore, take place at least initially only in the context of research, along the lines of OncorMed's policy. Visco advocated a direct research program to examine treatment options for women who test positive for deleterious *BRCA *mutations, to study what counseling is necessary and how to avoid discrimination in health insurance and employment. She optimistically recognized that:

"This is a time when we, as breast cancer activists, need the medical community to stand with us and recognize the need to do less, rather than more... We have a wonderful opportunity here: we can form a partnership between patient and physician and thoughtfully respond to this discovery... We ask that you take advantage of this standard of care, one that recognizes how little we know and is designed to get the answers." Frances Visco, on behalf of the National Breast Cancer Coalition [55].

In this statement, NBCC, as the pre-eminent organization for breast cancer activism, asserted its right to a place at the table with the scientists, policy makers, and corporate heads charged with implementing *BRCA *testing.

Mark Skolnick offered conflicting commentary in the same issue of the *Journal of Clinical Oncology*. His arguments directly countered Visco's, stating that to withhold the launch of the test, even to make time for the study recommended by Visco, would be to, "turn back the clock and ignore the ability to provide knowledge to women who seek it; to me this is unethical." Skolnick directly opposed the sentiments of NBCC and announced a release plan for *BRCA *testing that flaunted NBCC's suggestions (confirmed in an interview between the authors and Frances Visco). In the wake of Myriad's lawsuit, NBCC has gone on record in opposition to Myriad's patents, although neither NBCC nor FORCE is a direct party in the ongoing lawsuit (however, BCA is a party to the lawsuit against Myriad).

The role of the patents in these stories is ambiguous: the monopoly gave Myriad strong exclusive rights that bolstered its legal authority, and may have emboldened it. Or perhaps the patents had little to do with the degree to which the company abided by professional recommendations and related to disease advocacy organizations. By heeding Visco's recommendations in the beginning, Myriad might have mitigated some of the public outcry by forging alliances instead of fostering enmity. OncorMed, the firm that had licensed Mary-Claire King's genetic linkage patent and Michael Stratton's *BRCA *patents, had committed to a research program to build evidence for the utility of *BRCA *testing; Myriad made a different strategic choice. Since both firms had patents, it is clear patents did not drive the differing choices, although Myriad's business model was enabled by its patents and subsequent emergence as sole provider of *BRCA *testing with OncorMed's demise. It is hard to imagine Myriad could have been as successful in becoming the dominant US testing service, even in clinical research, without its patent rights, so patent exclusivity is a contributing cause. But the fact that other models for deploying patented genetic diagnostics can avoid the controversies associated with BRACAnalysis^® ^shows that business decisions about how to use exclusivity require separate attention in the causal network. Patents were necessary but not sufficient to construct the narrative of villainy associated with Myriad.

**Figure 1:**
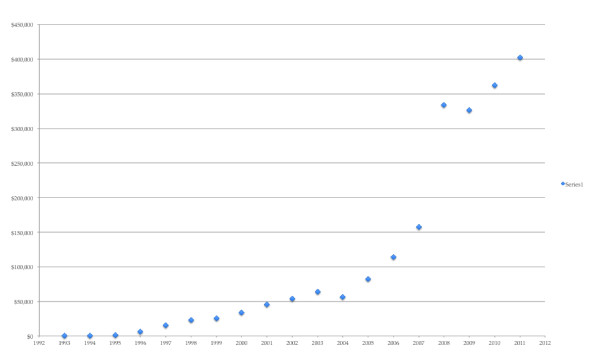
**Myriad Genetics revenue stream from 1993 to 2011**. Data derived from [66].

Myriad emerged from its patent battles with OncorMed with patent rights to *BRCA1*/*2 *and went on to turn a profit, while OncorMed did not survive. OncorMed's strategy may have bred less controversy, but Myriad is the firm that went on to survive as a business. Figure 1 demonstrates this by showing Myriad's revenue stream from 1993 to 2012.

The settlement between the companies may well have been driven by the perceived likely outcomes of patent litigation or the outcome of a patent interference proceeding. Outcomes from the private settlement suggest Myriad had the upper hand. OncorMed failed in its *BRCA *testing service despite its better relations with health professional organizations and advocacy organizations as a 'good citizen' vowing compliance with health professional standards; Myriad emerged with patent rights but poor relations with organizations representing its customer base of physicians and families at risk of breast and ovarian cancer. It is clear that from 2001 to 2012, Myriad's was the more successful business strategy, mainly because of its patent position.

Myriad's strategy now faces a transition in which ill will could have real business consequences. It faces four challenges: new technology, expiration of patents, possible invalidation of patent claims, and erosion of its proprietary database. As DNA sequencing costs drop, whole-genome and all-exome sequencing is coming into the cost range for sequencing just *BRCA1*/*2*. New competitors might sequence not only the two *BRCA *genes but also find mutations in other genes associated with breast and ovarian cancer [56] - or all genes - for more or less the same cost at Myriad's two-gene test. Myriad's broadest patents will begin to expire in 2014. The ongoing patent litigation has already invalidated Myriad's broadest patent claims - to methods for detecting mutations in *BRCA *genes. Its claims on short DNA fragments (for example, claims 5 and 6 of US 5,747, 282) are likely also invalid [57], and these are among the only claims that would preclude diagnostic testing, other than through PCR methods, which fall under claim 16 of US Patent 5,747,282. Since PCR amplification is unlikely to be a necessary step in future multi-gene sequencing strategies, Myriad's exclusivity in *BRCA *genetic testing could be undermined by the plummeting cost of multi-gene deep sequencing, all-exome sequencing, and whole-genome sequencing. Depending on the outcome of the pending case, Myriad could end up with only patents on specific mutations associated with cancer risk, or with no patents on naturally occurring sequences at all. (Myriad has patents on several specific mutations and methods for detecting them, some extending into at least 2028 [58].) Even if Myriad's patents are not valid, Myriad does have its proprietary database, but the value of that database will diminish as publicly funded research identifies disease-associated and neutral mutations. Moreover, if payers or health professionals demanded access to data to verify Myriad's determinations of cancer risk as a condition of payment, then Myriad's trade secret might no longer remain secret [59].

Myriad's 'diagnostic blockbuster' model has proven to be a $400 million per year business success. It does not follow, however, that it will withstand technological competition, patent expiration and litigation or determined public efforts to improve interpretation of *BRCA *mutations. In this way, like patented brand-name drugs, Myriad's blockbuster model is vulnerable to introduction of competition with patent expiry or technological competition. OncorMed's strategy might have produced a loyal customer base of those ordering and seeking *BRCA *testing; if Myriad's customers are only using its services grudgingly, and jump to competitors as soon as they can, then Myriad's business success could be evanescent. Whether Myriad's long-term business success depends on its exclusive patent rights will become more apparent over the next few years.

#### Genentech

In contrast to the public image of Myriad's corporate relations with prominent advocacy groups, Genentech has historically been hailed as a strong community partner, and an ally with the NBCC in developing Herceptin^® ^(this alliance has not survived differing positions on Avastin^® ^and drug pricing [60], however, so the alliance was strategic and contingent). Genentech's outreach to advocates after facing determined opposition is particularly instructive.

On 16 August 1995, the *San Francisco Weekly *ran an article on Genentech, entitled 'As they lay dying: cancer advocates rage at Genentech for withholding an experimental drug' [61]. This is not the kind of press a biotechnology firm wants in the morning paper. The article told of Marti Nelson, a breast cancer patient and advocate with BCA. Upon suspecting that she might be aided by treatment with Herceptin^®^, then in the early clinical trial stages, she pressed Genentech to provide her the medication on a 'compassionate use' basis. "Genentech refused to give Nelson HER-2/neu [sic]. She died on Nov. 9, 1994." A fellow advocate was quoted in this article, declaring, "How many women have to die on Genentech's doorstep before they do even one compassionate use for HER-2/neu [sic]... We are not going to let this rest. What Genentech is doing is really ugly." In the months leading up to the publication of that article, BCA had done anything but let it rest.

On 5 December 1994, advocates marched on the Genentech campus, demanding Herceptin^® ^access for compassionate use [30]. This got the attention of John Curd, Genentech's Immunology and Oncology Clinical Trials director (page 122 in [30]). Recognizing that he was caught between advocacy that was "not misguided" and the pragmatic corporate need to deny their wishes, he sought the help of Washington-based advocate Frances Visco [30]. As told in Robert Bazell's book, *Her-2*, Curd recalls Visco's advice to him: "Fran Visco said to me, 'John. I agree with you intellectually and scientifically. Compassionate use does not make a lot of sense. I'd like to see the data. But this is not an intellectual issue. This is an emotional and political issue. And politically, you have to have a compassionate use program'" [30]. With this collaboration was born a new approach to pharmaceutical development at Genentech: advocates were brought to the table.

In April 1995, advocates from many groups, including NBCC, were invited to help design the phase III trials. This new model of collaboration resulted in a markedly successful trial. Visco noted in retrospect that Genentech was the first pharmaceutical company that agreed to partner with breast cancer advocates, commenting that, "Genentech worked with us on all aspects of the trial, from protocol design, to outreach, to oversight. In particular, the company's agreeing to our request that investigators in specific communities partner with trained activists in facilitating accrual to the trial helped lead to record accrual"[62]. In October of 1999, Genentech was awarded the Corporate Leadership Award for their collaboration with NBCC, holding up this collaboration between advocates and industry as the standard for "a new model of cancer research" [14].

#### Different corporate responses to conflict with advocates

A critical difference between Genentech and Myriad was Genentech's open and systematic cultivation of collaborative and amicable relationships with advocacy groups representing their client base in response to early conflict. Both companies encountered conflict; Genentech made it productive while Myriad persisted with its initial business plan. The populations served by Genentech and Myriad overlapped to a significant extent. Media coverage of both conflicts includes very similar negative language alluding to companies favoring profit over patient access. The narratives differ at the point of resolution: Genentech made a deliberate effort to repair a poor relationship with BCA and turned to NBCC as an active partner, whereas Myriad did little to reconcile with FORCE or NBCC. Myriad has made efforts to create its own advocacy organizations, but these lack credibility precisely because they are corporate-affiliated, rather than collaborations with established, credible, national organizations.

Amicable and meaningful relationships with advocacy organizations dedicated to both patients and clinicians can matter to a company's success. This is not a simple matter; NBCC is not at all happy with Genentech's pursuit of the breast cancer indication for the highly expensive Avastin^®^. But no organization sued Genentech over its Herceptin^® ^patent, or even over Avastin^®^. BCA did join the suit against Myriad's patent, and NBCC and FORCE have made statements opposing Myriad's management of its *BRCA *patents [43,63] even if they did not join the plaintiffs in the case.

Genentech was wise, and ultimately more successful, to recognize the detrimental effect of a poor corporate image and the very practical benefit of an engaged constituency that could mobilize clinical research participation. Genentech moved forward with a collaborative approach to clinical trial implementation. Myriad's unapologetic decisions to test outside health professional recommendations and to risk alienating national organizations representing breast cancer advocates is where these stories fundamentally diverge [64].

Considering that both Genentech and Myriad had patent protection, we can conclude that the attribution of cause and the construction of venal narratives are to some degree misdirected at patents. In health care, company strategy is inherently bound to the consumers that it serves, regardless of patent protection. When the product being sold is so directly related to health and wellbeing, the stakes and standards for benevolent corporate behavior are raised. As Zoe Christopher, a resource liaison for BCA stated in her contribution to the American Civil Liberties Union's *Take Back your Genes *public campaign, "I take back my genes so that profit will not be at the expense of women's lives" [65]. We speculate that it is primarily Myriad's failure to acknowledge this, not just their patent protection, that has contributed to the problematic escalation of the debate. 'Profits over patients' is a common refrain in health care services, and by no means restricted to BRACAnalysis^® ^or any other particular product; neither is it confined to products and services covered by patents. 'Patents over patients' is a verse of the general 'profit' refrain. It is nonetheless instructive that when Genentech encountered opposition from activists, it recalibrated its strategy for developing Herceptin^®^; when Myriad encountered similar opposition, it largely ignored it, countered it, or attributed it to misinformation or lack of understanding. What Myriad did not do was change its plan, or even its rhetoric.

## Summary

*Amicus curiae *and famous co-discoverer of the DNA double helix James Watson concluded his *amicus *brief by quoting his own words: "The Human Genome Project... is as precious a body of knowledge as humankind will ever acquire, with a potential to speak to our most basic philosophical questions about human nature, for purposes of good and mischief alike" [66]. In both the scientific and political aftermath of the Human Genome Project, Watson's assertion has largely held true. As the Myriad case highlights, the social, ethical, and legal implications of genomics are continually emerging, even decades after the discovery of DNA. For as much as research has discovered and promises to discover, inexperience abounds and these questions linger: the idealized promise of a simple correlation between genotype and phenotype is gradually being replaced by a more sophisticated understanding of the science and the disciplinary maturity to accept what will perhaps always remain a mystery, the complicated relation between genotype, biology and health. By analogy, this history also suggests a need for sophistication of how the accompanying social narratives of that science come to be constructed. If this mystery exists in the science, surely we can expect the same degree of uncertainty in the ethical and legal ramifications of that science, including the way business strategies are crafted and adjusted (or not).

Several strategic decisions contributed to Myriad's abysmal corporate image. By comparing Myriad's decisions to those of Genentech and OncorMed, it becomes apparent that the story is not just about patents. There are many points of contention, and the full weight of Myriad's controversial business model should not be attributed to patents alone. Arguments about patentable subject matter are playing out between the American Civil Liberties Union and its plaintiffs and Myriad and the defendants in the case before the US Supreme Court, but our purpose here is to stress that the Myriad case is an exceptional one, and not the rule. The legal debate has focused solely on patents, but Myriad was largely sued because of its business model: patents provided a legal scapegoat. While many would agree that Myriad should be held accountable for the practices that patents did indeed allow them to pursue, the legal consequences are ultimately not only aimed at the vehicle driving these practices, but also at the public road this vehicle is utilizing, the patent system. In short, this comparative case study shows it is not only - or even mainly - about the patents.

The risk of a patent-centered narrative is that the crucial nuances of this story will be lost in monomaniacal attention to whether genes can be patented, to the neglect of business practices that are equally to blame. The social issues that have accompanied the vigorous, often acrimonious, debate about gene patents have both legal and moral elements, and that debate will go on, regardless of court decisions in the ongoing *BRCA *lawsuit.

The public debate has been impoverished in two respects, by assuming: (1) that the cause of the problems underlying the debate are inherently due to patents; and (2) that eliminating gene patents will solve those problems. Patents contribute and enable business practices, but many genes have been patented that did not lead to the same problems. The story of *BRCA *testing is about business decisions that did indeed rest on enforcing exclusive rights in the US market - but the story is also about failing to include advocacy organizations and disregarding health professional standards. Patents matter, but so do business practices.

## Abbreviations

BCA: Breast Cancer Action; BIC: Breast Cancer Information Core; CAFC: Court of Appeals for the Federal Circuit; FDA: Food and Drug Administration; FORCE: Facing our Risk of Cancer Empowered; NBCC: National Breast Cancer Coalition; NCI: National Cancer Institute; SEC: Securities and Exchange Commission; USPTO: US Patent and Trademark Office.

## Competing interests

LB declares no competing interests. RC-D is on the *BMJ *list of experts who agree they do not have "any financial support in any form from pharmaceutical or medical device manufacturers during the past five years and that they do not have other affiliations or financial involvements that would present a conflict of interest. A three member board decides whether to accept applicants" [67]. He accordingly declares no competing interests for this article.

## Authors' contributions

RC-D conceived of the paper, helped to draft and edit the manuscript, and participated in its design. ALB helped to draft and edit the manuscript, and participated in its design. Both authors read and approved the final manuscript.

## Authors' information

RC-D is research professor and director of Genome, Ethics, Law & Policy, Institute for Genome Sciences & Policy and Sanford School of Public Policy at Duke University, Durham, NC, USA. ALB is a research aide in Genome, Ethics, Law & Policy at Duke University, where she is part of the Center for Public Genomics.
